# A Case of Non-diabetic Hypoglycemia in the Elderly: Insulin Autoimmune Syndrome

**DOI:** 10.7759/cureus.108773

**Published:** 2026-05-13

**Authors:** Niveda Nagendran, Ramya Venkatesan, Mohini Singh, Ramakrishnan S R

**Affiliations:** 1 General Medicine, Sri Ramachandra Institute of Higher Education and Research, Chennai, IND

**Keywords:** autoimmune hypoglycemia, endogenous hyperinsulinemia, hirata disease, insulin autoantibodies, recurrent hypoglycemia

## Abstract

Insulin autoimmune syndrome (IAS), also known as Hirata syndrome, is a rare cause of spontaneous hyperinsulinemic hypoglycemia, most commonly reported in Asian populations, characterized by the presence of insulin autoantibodies in individuals without prior exposure to exogenous insulin. We report the case of a 71-year-old woman who presented with recurrent postprandial hypoglycemic episodes associated with neuroglycopenic symptoms. There was no history of insulin use, oral hypoglycemic agents, or exposure to drugs known to precipitate IAS. Biochemical evaluation demonstrated marked hyperinsulinemia (>1000 µIU/mL), elevated C-peptide levels, an insulin-to-C-peptide ratio >1, and positive insulin autoantibodies, confirming the diagnosis. Anti-thyroid peroxidase antibodies were elevated with normal thyroid function, suggesting an underlying autoimmune predisposition. She was treated with corticosteroids and dietary modification, resulting in complete resolution without recurrence. Steroids were tapered and discontinued over three months with sustained remission. IAS should be considered in cases of non-diabetic hypoglycemia, and early diagnosis with appropriate management can prevent complications.

## Introduction

Hypoglycemia is a potentially life-threatening condition that requires prompt evaluation to identify the underlying etiology. While it is most commonly associated with diabetes, spontaneous hypoglycemia in non-diabetic individuals necessitates evaluation for endogenous hyperinsulinemic states, including rare autoimmune conditions such as insulin autoimmune syndrome (IAS).

IAS, first described by Hirata, is characterized by the presence of insulin autoantibodies in individuals without prior exposure to exogenous insulin. The syndrome may occur spontaneously or in association with viral illnesses and exposure to drugs, particularly sulfhydryl-containing compounds such as methimazole, alpha-lipoic acid, and captopril. It is most frequently reported in Japanese and other Asian populations and typically presents in the seventh decade of life in females and earlier in males [1,2]. Despite increasing recognition, IAS remains under-reported due to difficulty in performing antibody testing [1].

IAS is clinically important because it can mimic insulinoma and other causes of hypoglycemia, but it differs in pathophysiology and management. Early recognition avoids unnecessary investigations and surgeries and enables appropriate treatment.

## Case presentation

A woman in her early 70s with a history of hypertension on amlodipine and bronchial asthma on intermittent reliever therapy presented with a one-week history of recurrent episodes of drowsiness associated with increased sweating and palpitations relieved immediately on food intake. There was no history of seizures, loss of consciousness, or focal neurological deficits, prior history of diabetes mellitus, reduced oral intake, significant weight loss, prior gastric surgery, or family history of autoimmune disease. Initial evaluation revealed hypoglycemia during symptomatic episodes, following which further evaluation for the etiology of hypoglycemia was undertaken. Neuroimaging was unremarkable.

History confirmed no prior exposure to exogenous insulin, oral hypoglycemic agents, anti-thyroid drugs, alpha-lipoic acid supplementation, herbal medications, health supplements, or sulfhydryl-containing drugs known to precipitate IAS. Evaluation excluded other causes of hypoglycemia, including hepatic failure, renal failure, cardiac failure, sepsis, and adrenal insufficiency, based on clinical assessment and laboratory investigations.

The patient fulfilled Whipple's triad, with documented hypoglycemia, corresponding symptoms, and resolution following glucose administration [2]. Despite severe hypoglycemia (lowest recorded value 32 mg/dL; hypoglycemia defined as plasma glucose <70 mg/dL and clinically significant hypoglycemia as <54 mg/dL), she did not develop seizures, highlighting variability in neuroglycopenic thresholds [3]. Recurrent hypoglycemic episodes raised concern for hypoglycemia-associated autonomic failure (HAAF), which may blunt typical adrenergic warning symptoms [3].

During hospitalization, she experienced recurrent predominantly postprandial hypoglycemic episodes. Critical sampling during these episodes demonstrated plasma glucose of 51 mg/dL, markedly elevated insulin (>1000 µIU/mL), and elevated C-peptide (21.69 ng/mL), confirming endogenous hyperinsulinemia. The insulin-to-C-peptide ratio was greater than 1, supporting a diagnosis of IAS [2]. Key biochemical findings are summarized in Table 1.

**Table 1:** Key biochemical findings Values represent laboratory measurements obtained during the two-hour postprandial hypoglycemic episode. HbA1c: glycated hemoglobin

Parameter	Value	Reference range	Interpretation
Plasma glucose	51 mg/dL	70-100 mg/dL (fasting)	Hypoglycemia
Insulin	>1000 µIU/mL	2-25 µIU/mL (fasting)	Markedly elevated
C-peptide	21.69 ng/mL	0.5-2.0 ng/mL (fasting)	Markedly elevated consistent with endogenous hyperinsulinism
HbA1c	5.4%	4.0-5.6%	Normal
8 am cortisol	14.96 µg/dL	5-25 µg/dL	Adequate

Urine ketones were negative, supporting hyperinsulinemic hypoglycemia. Glycated hemoglobin (HbA1c) was within normal limits (5.4%), indicating the absence of chronic hyperglycemia. Insulin autoantibodies measured using an immunoassay-based serological method were markedly elevated, confirming the diagnosis [2]. Additional investigations are summarized in Table 2.

**Table 2:** Additional investigations Summary of ancillary investigations performed to evaluate the etiology of hypoglycemia. anti-TPO: anti-thyroid peroxidase antibody; TSH: thyroid-stimulating hormone

Parameter	Finding
Urine ketones	Negative
Anti-TPO	Elevated
Thyroid function testing (fT3, fT4, TSH)	Normal
Ultrasound of the abdomen	No pancreatic lesion
Blood/urine culture	Sterile

Anti-thyroid peroxidase antibodies were elevated; however, thyroid function tests were normal, and there was no history of anti-thyroid drug exposure, suggesting an underlying autoimmune predisposition without features of autoimmune polyglandular syndrome.

Ultrasonography of the abdomen revealed no pancreatic mass lesion. There was no evidence of infection, and blood and urine cultures were sterile. There was no clinical or laboratory evidence suggestive of plasma cell dyscrasia, including multiple myeloma or monoclonal gammopathy.

The patient was initiated on oral prednisolone (1 mg/kg/day) along with dietary modification emphasizing frequent complex carbohydrate intake. She showed rapid clinical improvement with complete resolution of hypoglycemic episodes. Corticosteroids were gradually tapered and discontinued over a period of three months. At follow-up, she remained asymptomatic with no recurrence of hypoglycemia.

## Discussion

IAS is a rare cause of endogenous hyperinsulinemic hypoglycemia characterized by circulating insulin autoantibodies in individuals without prior exposure to exogenous insulin. Although initially described predominantly in Japanese populations, IAS is increasingly recognized worldwide [2]. The peak age of onset is typically in the seventh decade, consistent with our case [1,2].

IAS may occur spontaneously or be triggered by drugs, particularly sulfhydryl-containing compounds [2]. Our patient had no identifiable drug exposure, suggesting a spontaneous form of IAS.

The hallmark of IAS is the presence of insulin autoantibodies with high binding capacity and low affinity [2]. Following insulin secretion, large amounts of circulating insulin bind to these antibodies, resulting in transient hyperglycemia. Due to their low affinity, these insulin-antibody complexes dissociate unpredictably, releasing free insulin and resulting in postprandial hypoglycemia. This biphasic mechanism explains the paradoxical coexistence of hyperinsulinemia and hypoglycemia in IAS [2].

The presence of anti-thyroid peroxidase antibodies in our patient suggests an underlying autoimmune predisposition, consistent with the pathophysiology of IAS, although the criteria for autoimmune polyglandular syndrome were not fulfilled [3]. Uchigata et al. described the clinical and epidemiological features of IAS in Japan, where the condition is more commonly reported [4]. Cappellani et al. further highlighted the heterogeneous clinical presentation and variable biochemical severity seen across published cases [2].

Unlike insulinoma, IAS is characterized by the triad of markedly elevated circulating insulin levels, positive insulin autoantibodies, and absence of radiological evidence of pancreatic lesions. Similar to previously reported cases, this patient presented with adrenergic manifestations of hypoglycemia and elevated insulin autoantibodies in the absence of insulin therapy.

IAS should be suspected in patients presenting with spontaneous hypoglycemia, elevated insulin and C-peptide levels, absence of exogenous insulin use, and positive insulin autoantibodies [2]. Differentiation from insulinoma is crucial, as both conditions present with endogenous hyperinsulinemic hypoglycemia. However, IAS is characterized by markedly elevated insulin levels, with disproportionately high insulin autoantibodies, absence of pancreatic lesions on imaging, and a predominantly postprandial pattern of hypoglycemia, whereas insulinoma typically presents with fasting hypoglycemia and identifiable pancreatic tumors [5].

Failure to recognize IAS may lead to unnecessary invasive investigations or surgical interventions, underscoring the importance of considering this diagnosis early in the evaluation of unexplained hypoglycemia.

Management includes dietary modification and corticosteroid therapy, which reduces antibody production [2]. Most patients improve with dietary modification, avoidance of precipitating drugs, and conservative management, although corticosteroids and immunosuppressive therapy may be required in severe or persistent cases [4,6]. Our case highlights the importance of considering IAS in elderly patients presenting with unexplained postprandial hypoglycemia, even in the absence of identifiable triggers.

## Conclusions

IAS is an important and underrecognized cause of spontaneous hypoglycemia. It should be considered in patients with hyperinsulinemic hypoglycemia without exogenous insulin exposure. Recognition of its characteristic pathophysiology and appropriate diagnostic evaluation are essential to avoid unnecessary investigations. Early treatment with corticosteroids and dietary modification is effective and associated with a good prognosis.
